# NKX6.1 Represses Tumorigenesis, Metastasis, and Chemoresistance in Colorectal Cancer

**DOI:** 10.3390/ijms21145106

**Published:** 2020-07-19

**Authors:** Hsin-Hua Chung, Chun-Te Lee, Je-Ming Hu, Yu-Ching Chou, Ya-Wen Lin, Yu-Lueng Shih

**Affiliations:** 1 Graduate Institute of Medical Sciences, National Defense Medical Center, No.161, Sec.6, Minquan East Rd., Neihu District, Taipei 11490, Taiwan; im.andrew7@gmail.com (H.-H.C.); jeminghu@gmail.com (J.-M.H.); ndmc.yawen@msa.hinet.net (Y.-W.L.); 2 Division of Urological Surgery, Department of Surgery, Tri-Service General Hospital Songshan Branch, No.131, Jiankang Rd., Songshan District, Taipei 10581, Taiwan; g559100016@gmail.com; 3 Division of Colorectal Surgery, Department of Surgery, Tri-Service General Hospital, National Defense Medical Center, No.325, Sec.2, Chenggong Rd., Neihu District, Taipei 11490, Taiwan; 4 School of Public Health, National Defense Medical Center, No.161, Sec.6, Minquan East Rd., Neihu District, Taipei 11490, Taiwan; trishow@mail.ndmctsgh.edu.tw; 5 Department and Graduate Institute of Microbiology and Immunology, National Defense Medical Center, No.161, Sec.6, Minquan East Rd., Neihu District, Taipei 11490, Taiwan; 6 Graduate Institute of Life Sciences, National Defense Medical Center, No.161, Sec.6, Minquan East Rd., Neihu District, Taipei 11490, Taiwan; 7 Division of Gastroenterology, Department of Internal Medicine, Tri-Service General Hospital, National Defense Medical Center, No. 325, Sec. 2, Chenggong Rd., Neihu District, Taipei 11490, Taiwan

**Keywords:** NK6 homeobox 1 (NKX6.1), colorectal cancer (CRC), tumor suppressor, metastasis, epithelial-to-mesenchymal transition (EMT), chemoresistance

## Abstract

Accumulating evidence suggests that NKX6.1 (NK homeobox 1) plays a role in various types of cancer. In our previous studies, we identified *NKX6.1* hypermethylation as a promising marker and demonstrated that the NKX6.1 gene functions as a metastasis suppressor through the epigenetic regulation of the epithelial-to-mesenchymal transition (EMT) in cervical cancer. More recently, we have demonstrated that *NKX6.1* methylation is related to the chemotherapy response in colorectal cancer (CRC). Nevertheless, the biological function of NKX6.1 in the tumorigenesis of CRC remains unclear. In this study, we showed that NKX6.1 suppresses tumorigenic and metastatic ability both in vitro and in vivo. NKX6.1 represses cell invasion partly through the modulation of EMT. The overexpression of NKX6.1 enhances chemosensitivity in CRC cells. To further explore how NKX6.1 exerts its tumor-suppressive function, we used RNA sequencing technology for comprehensive analysis. The results showed that differentially expressed genes (DEGs) were mainly related to cell migration, response to drug, transcription factor activity, and growth factor activity, suggesting that these DEGs are involved in the function of NKX6.1 suppressing cancer invasion and metastasis. Our results demonstrated that NKX6.1 functions as a tumor suppressor partly by repressing EMT and enhancing chemosensitivity in CRC, making it a potential therapeutic target.

## 1. Introduction

Colorectal cancer (CRC) is one of the leading causes of cancer incidence and mortality worldwide [1]. Recurrence and metastasis are still the main reasons for death from CRC, even though advances in strategies for early diagnosis and treatment have been made [2]. While the first-line chemotherapeutic treatment for CRC, 5-fluorouracil (5-FU) in combination with oxaliplatin (L-OHP) or irinotecan (CPT-11), is generally effective, most patients ultimately evolve resistance to these chemotherapeutic agents [3,4]. Drug resistance can result in a reduction of drug accumulation within the cell by reducing uptake, enhancing efflux, or affecting membrane lipids such as ceramide [5,6]. The resistance to apoptosis due to a regulated cell cycle and enhanced DNA damage repair may also contribute to drug resistance [7,8]. In addition, epigenetic modifications may play significant roles in mediating drug resistance in certain types of cancer [9]. To develop effective therapeutic strategies for CRC patients who do not respond well to such treatments, understanding of the mechanisms by which recurrence and drug resistance occur in CRC is urgently needed.

*NKX6-1* (NK homeobox 1) is also known as *NKX6.1* and *NKX6A*. NKX6.1 encodes a homeobox-containing protein that belongs to the NKX family of transcription factors with a DNA binding domain. *NKX6.1* was initially identified in Drosophila pancreatic β-cells, which regulates insulin-secreting β-cell differentiation by binding to and activating its own promoter while simultaneously widely repressing the activity of other genes, such as insulin [10]. NKX6.1 plays a crucial role in the neural development and differentiation of pancreatic cells [11,12,13,14]. The NKX family of genes has been found to play a role in various types of cancer. It has been reported that NKX2.1 functions as an oncogene and is correlated with lung cancer [15,16,17]. NKX3.1 functions as a negative regulator of epithelial cell growth in prostate tissue and is associated with prostate tumor progression [18,19]. In our previous studies, we identified *NKX6.1* hypermethylation as a promising marker and demonstrated that the NKX6.1 functions as a metastasis suppressor in cervical cancer [20,21,22,23]. In addition to our reports, the differential methylation of *NKX6.1* has also been observed in various types of cancer including B-cell lymphoma [24], acute lymphoblastic leukemia [25], astrocytoma [26] and gastric cancer [27,28]. Moreover, we recently demonstrated that *NKX6.1* methylation was an independent indicator of 5-year disease-free survival in stage II CRC patients receiving adjuvant chemotherapy [29,30]. Our findings suggested that NKX6.1 hypermethylation is related to the chemotherapy response and has medical potential in optimizing the selection of therapy. Nevertheless, the biological function of NKX6.1 in the tumorigenesis of CRC remains unclear.

The epithelial-to-mesenchymal transition (EMT) is known to not only occur in embryonic development but also contribute to cancer progression [31,32]. During the EMT process, the coupling of an induction of mesenchymal markers and inhibition of epithelial markers is commonly observed [32,33]. It is characterized by a loss of cell polarity and cell adhesion, inhibition of E-cadherin expression, and increased cell motility. Simultaneously, the features of epithelial cells such as apical-basal polarity morphological and molecular changes are transformed to mesenchymal characteristics, such as fibroblastic morphology, increased vimentin and N-cadherin expression, enhanced migration and invasion capacities, thus causing cancer cell metastasis [34]. Tumor metastasis is a complex process that comprises the detachment of cancer cells from the basal layer through EMT and proceeds to invasion, intravasation, circulation into blood vessels, extravasation, and finally metastasis to a distant secondary organ [35]. Several transcription factors, including SNAIL, SLUG, ZEB1, and TWIST, can suppress E-cadherin and enhance mesenchymal markers directly or indirectly [36,37]. The binding of the zinc finger transcription factor SNAIL to the E-box elements of the E-cadherin promoter can repress the transcription of E-cadherin [38]. SLUG, a member of the SNAIL family [39], as well as ZEB1 [40], can repress the transcription of E-cadherin, thereby promoting the dissociation of cell adhesion, and therefore inducing cell invasion and migration. TWIST can induce EMT; it indirectly represses the transcription of E-cadherin, which is an indirect repressor of the E-cadherin promoter [41]. More recently, we have demonstrated that NKX6.1 suppresses tumor metastasis through the epigenetic regulation of EMT. NKX6.1 could recruit the BAF155 coactivator to enhance the mRNA level of E-cadherin and recruit the RBBP7 (retinoblastoma binding protein 7) corepressor to repress that of vimentin in cervical cancer [20]. However, whether NKX6.1 affects the EMT process in CRC is still unknown.

The current study focused on investigating the role of NKX6.1 in CRC tumorigenesis, migration, and invasion. Our data showed that NKX6.1 functions as a tumor suppressor partly through the repression of EMT. Moreover, the overexpression of NKX6.1 enhances the chemosensitivity of CRC cell lines. These data suggested that NKX6.1 could be a potential therapeutic target in advanced CRC.

## 2. Results

### 2.1. Overexpression of NKX6.1 Inhibits the Transformation, Migration, and Invasion of CRC Cells

**Figure 1:**
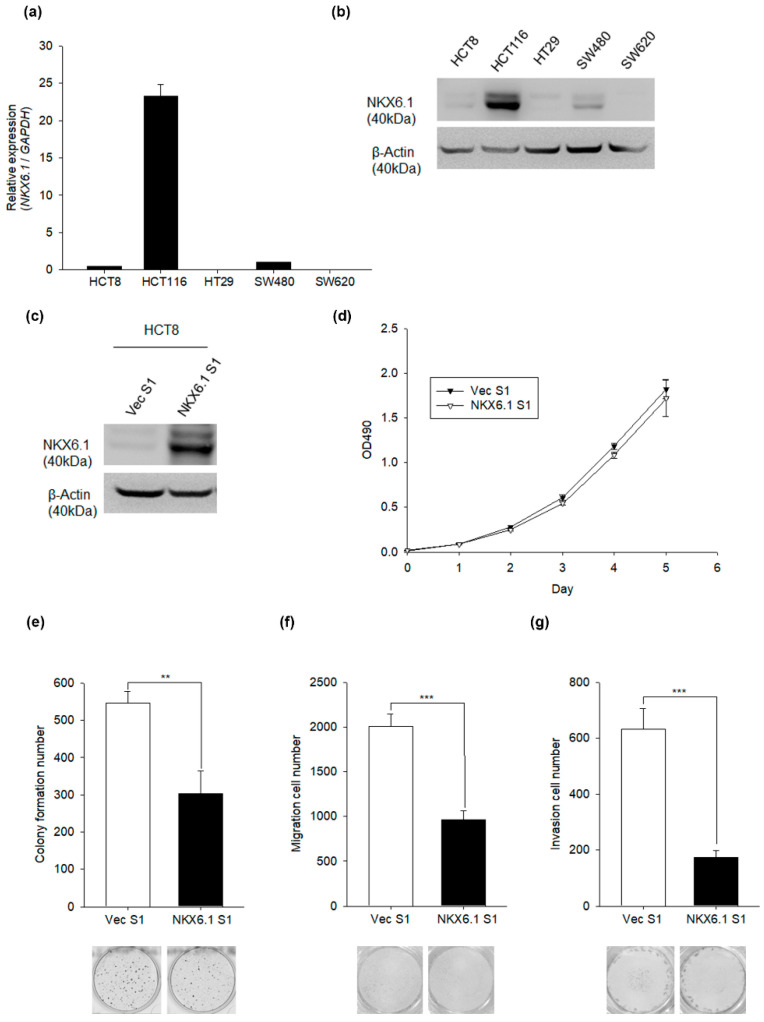
Overexpression of NKX6.1 (NK homeobox 1) represses the transformation, migration, and invasion of colorectal cancer (CRC) cells in a constitutive expression system. (**a**) NKX6.1 RNA levels were determined by qRT-PCR in human CRC cell lines. GAPDH was used as an internal control. (**b**) NKX6.1 protein expression was determined by Western blotting using an anti-NKX6.1 antibody in human CRC cell lines. β-Actin was used as an internal control. (**c**) Expression of NKX6.1 in HCT8 cells infected with lentiviruses harboring pLAS2.1-NKX6.1 (NKX6.1 S1) or the empty vector (Vec S1) were analyzed by Western blotting analysis using an anti-NKX6.1 antibody. β-Actin was used as an internal control. (**d**) Cell proliferation (MTS, 3-(4,5-dimethylthiazol-2-yl)-5-(3-carboxymethoxyphenyl)-2-(4-sulfophenyl)-2H-tetrazolium), (**e**) anchorage-independent growth assays (AIG), (**f**) transwell migration assays, and (**g**) Matrigel invasion assays were performed using HCT8 cells expressing NKX6.1 or the empty vector. The absorbance values, colony formation number, migration cell number, and invasion cell number are presented as the mean ± SD (analyzed by unpaired two-tailed *t*-test). ** *p* < 0.01 and *** *p* < 0.001.

The epigenetic silencing of NKX6.1 has been reported in CRC cells [29,30] but not in normal colon tissues. First, we examined the RNA level and protein expression of NKX6.1 in five CRC cell lines and normal colon tissues. Both RNA and the protein expression of NKX6.1 were downregulated in HCT8, HT29, SW480, and SW620 cell lines compared with HCT116 cell lines (Figure 1a,b) (Figure S1). To explore the biological function of NKX6.1 in CRC cells in vitro, we overexpressed NKX6.1 in HCT8 cells (Figure 1c) (Figure S2). Ectopic expression of NKX6.1 did not affect cell viability (Figure 1d), but it did significantly inhibit colony formation (Figure 1e). Furthermore, the overexpression of NKX6.1 also decreased the number of migrating and invading cells (Figure 1f,g).

**Figure 2:**
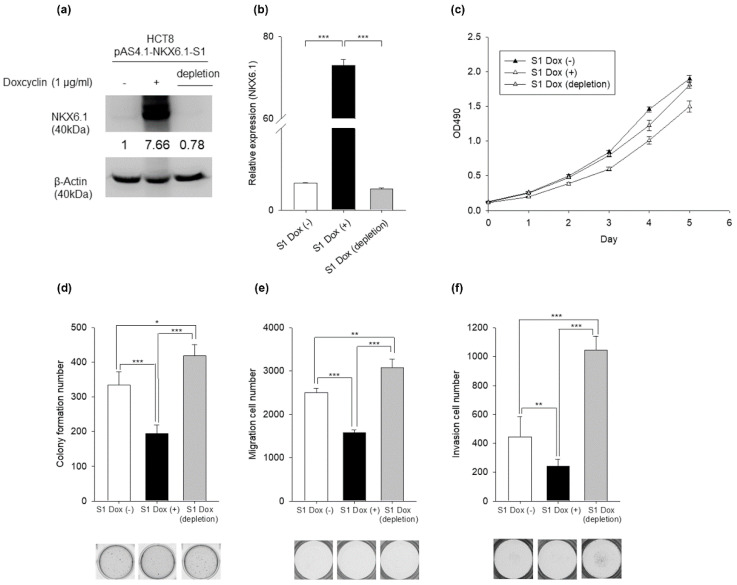
Overexpression of NKX6.1 represses the transformation, migration, and invasion of CRC cells in an inducible expression system. NKX6.1 overexpression in HCT8 cell lines infected with lentiviruses harboring pAS4.1-NKX6.1 (pAS4.1-NKX6.1-S1). (**a**,**b**) Dox (1 μg/mL)-inducible NKX6.1 expression was established in HCT8 cells and assessed by Western blotting analysis and qRT-PCR analysis. β-Actin was used as an internal control. GAPDH was used as an internal control. The numbers in the Western blots represent the ratios of targets to the internal control. (**c**) Cell proliferation (MTS), (**d**) colony formation, (**e**) migration, and (**f**) invasion assays were performed using HCT8 cells. The results are presented as the mean ± SD (analyzed by unpaired two-tailed *t*-test). * *p* < 0.05, ** *p* < 0.01, and *** *p* < 0.001.

To further investigate the suppressive effects of NKX6.1 on carcinogenesis in CRC, an inducible NKX6.1 expression system in HCT8 cells was established. Then, NKX6.1 was induced by 1 μg/mL doxycycline (Dox), and the depletion group was achieved by the withdrawal of Dox after Dox induction. These data confirmed that the induction and withdrawal of Dox modulated NKX6.1 mRNA and protein levels by using Western blotting and quantitative reverse-transcription-PCR (qRT-PCR) analyses (Figure 2a,b) (Figure S3). We assessed whether the induction of NKX6.1 affected cell viability, transformation, and invasion after induction by Dox for 3 days. The data showed that the cell viability of NKX6.1-overexpressing and NKX6.1-depleting cells was similar to that of the controls (Figure 2c). The overexpression of NKX6.1 suppressed colony formation (Figure 2d), migration, and invasion (Figure 2e,f). Upon the depletion of NKX6.1 by Dox withdrawal, the suppression of colony formation, migration, and invasion was reversed in the NKX6.1-depleted group. These results were consistent with the data obtained in cells constitutively expressing NKX6.1. Taken together, these results demonstrated that NKX6.1 suppresses the transformation, migration, and invasion of CRC.

### 2.2. Knockdown of NKX6.1 Promotes the Transformation, Migration, and Invasion of CRC Cells

**Figure 3:**
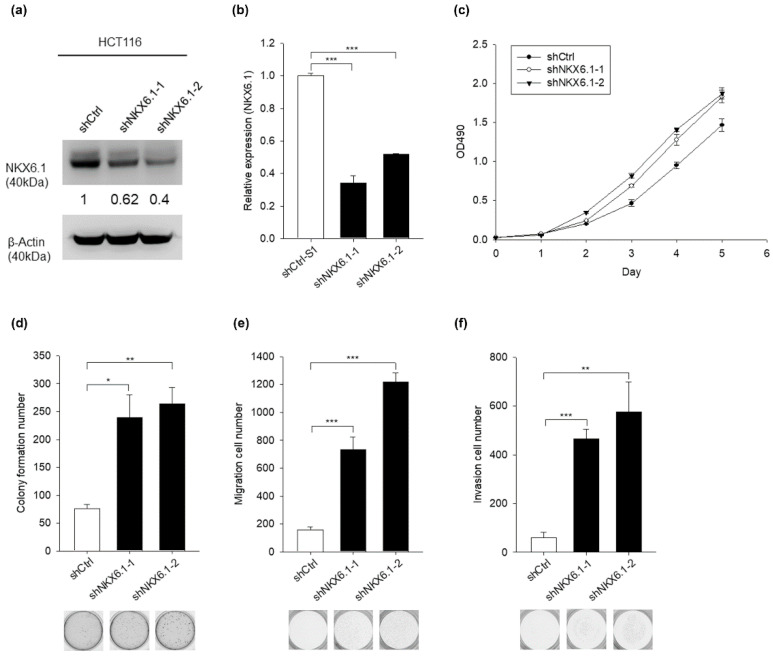
Knockdown of NKX6.1 promotes the transformation, migration, and invasion of CRC cells. (**a**) NKX6.1 protein expression was determined by Western blotting in HCT116 cell lines stably transfected with control shRNA (shCtrl) or NKX6.1 shRNA (shNKX6.1-1, shNKX6.1-2). β-Actin was used as an internal control. The numbers in the Western blots represent the ratios of targets to the internal control. (**b**) NKX6.1 RNA levels were determined by qRT-PCR in HCT116 cell lines stably transfected with control shRNA (shCtrl) or NKX6.1 shRNA (shNKX6.1-1, shNKX6.1-2). GAPDH was used as an internal control. (**c**) Cell proliferation (MTS), (**d**) colony formation, (**e**) migration, and (**f**) invasion assays were performed using HCT116 cells. The results are presented as the mean ± SD (analyzed by unpaired two-tailed *t*-test). * *p* < 0.05, ** *p* < 0.01, and *** *p* < 0.001.

We next investigated whether the knockdown of NKX6.1 influences the malignant phenotypes of CRC cells. We transfected HCT116 cells with two small hairpin RNAs (shRNAs) that target distinct coding sequences of NKX6.1 (shNKX6.1-1 and shNKX6.1-2) or a control shRNA (shCtrl). The two NKX6.1-shRNA transfected cells showed the downregulation of NKX6.1 expression at mRNA and protein levels compared with the nonsilencing control (shCtrl) in HCT116 cells (Figure 3a,b) (Figure S4). There was no significant difference in cell viability between NKX6.1-knockdown cells and nonsilencing control cells (Figure 3c). The knockdown of NKX6.1 significantly promoted the cell transformation, migration, and invasion of HCT116 cells (Figure 3d−f). Taken together, these results demonstrated that the knockdown of NKX6.1 enhances the transformation, migration, and invasion of CRC cells.

### 2.3. Overexpression of NKX6.1 Represses Tumorigenicity and Metastasis in Xenograft Mouse Models

**Figure 4:**
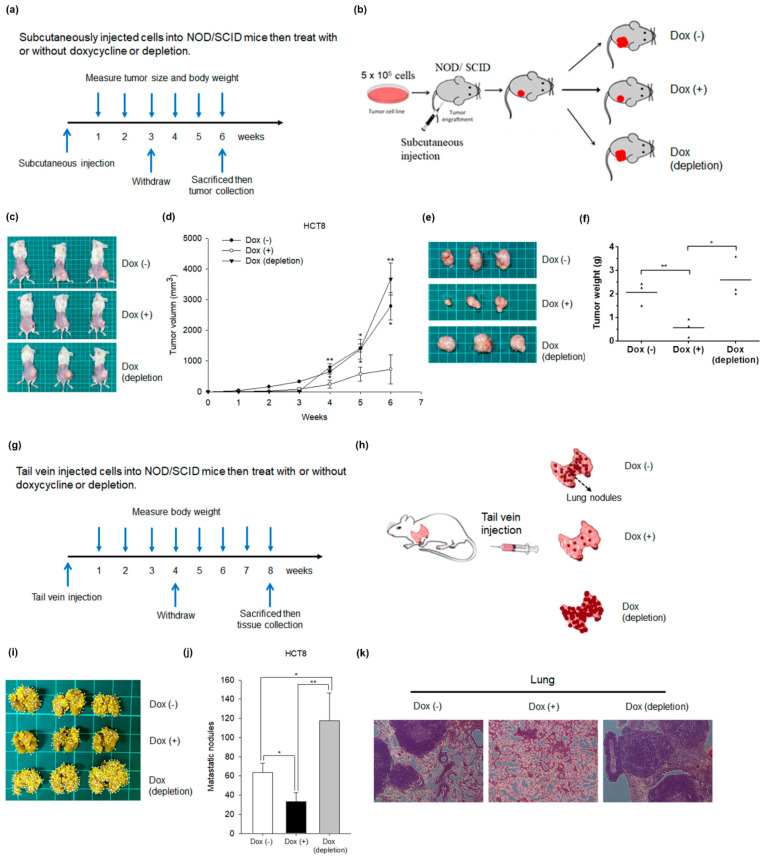
Overexpression of NKX6.1 represses tumorigenesis and metastasis in xenograft mouse models. HCT8 cell lines infected with lentiviruses harboring pAS4.1-NKX6.1 (NKX6.1 S1) were subcutaneously injected into NOD/SCID (nonobese diabetic severe-combined immunodeficiency) mice. (**a**) The detailed manipulations of animal experiments are illustrated. (**b**) Schematic demonstrating the tumorigenesis assay model in vivo. The tumor growth and curve (**c**,**d**) and tumor weights and curve (**e**,**f**) of the NKX6.1-expressing cells (Dox(+)) were compared with controls (Dox(-)) and Dox depletion. HCT8 cell lines infected with lentiviruses harboring pAS4.1-NKX6.1 (NKX6.1 S1) was injected into the tail vein of NOD/SCID mice. (**g**) The detailed manipulations of the animal experiment are illustrated. (**h**) Schematic demonstrating the metastasis assay model in vivo. (**i**) The images are lungs excised from the mice; the arrows indicate lung nodules. (**j**) Lung nodule number. (**k**) The images are hematoxylin and eosin (H&E) staining of lungs (original magnification, ×200) from the mice. The values are expressed as the mean ± SD (analyzed by unpaired two-tailed *t*-test). * *p* < 0.05 and ** *p* < 0.01.

To elucidate whether the overexpression of NKX6.1 affects tumorigenicity in vivo, we subcutaneously injected 5 × 10^5^ HCT8 cells with inducible NKX6.1 expression into the right flanks of NOD/SCID (nonobese diabetic severe-combined immunodeficiency) mice. We grouped the NOD/SCID mice into control (Dox free), NKX6.1-expressing (added 0.2 μg/mL Dox in the drinking water), and NKX6.1-depleting (Dox withdrawal after Dox induction for three weeks) groups (Figure 4a,b). Six weeks after injection, the tumor mass was smaller in the NKX6.1-expressing group than in the control group. The tumor growth rate was also decreased in the NKX6.1-expressing group. Furthermore, the suppressive effect on tumor mass and tumor growth rate was reversed in NKX6.1-depleted cells (Figure 4c−f).

To further investigate the effect of NKX6.1 on metastasis in vivo, we injected HCT8 cells with inducible NKX6.1 expression into mice via the tail vein. We grouped the NOD/SCID mice into control (Dox free), NKX6.1-expressing (added 0.2 μg/mL Dox in the drinking water), and NKX6.1-depleting (Dox withdrawal after Dox induction for four weeks) (Figure 4g,h). Eight weeks after injection, we found that the NKX6.1-expressing mice had fewer metastatic lung nodules than the control group. Moreover, the metastatic lung nodules in the NKX6.1-depleted mice were increased compared with those in the NKX6.1-expressing group (Figure 4i−k). NKX6.1 suppressed tumor growth and metastasis in xenograft models. Taken together, these data demonstrated that NKX6.1 plays critical roles in tumor formation and metastasis.

### 2.4. Knockdown of NKX6.1 Promotes Tumorigenesis and Metastasis in Xenograft Mouse Models

**Figure 5:**
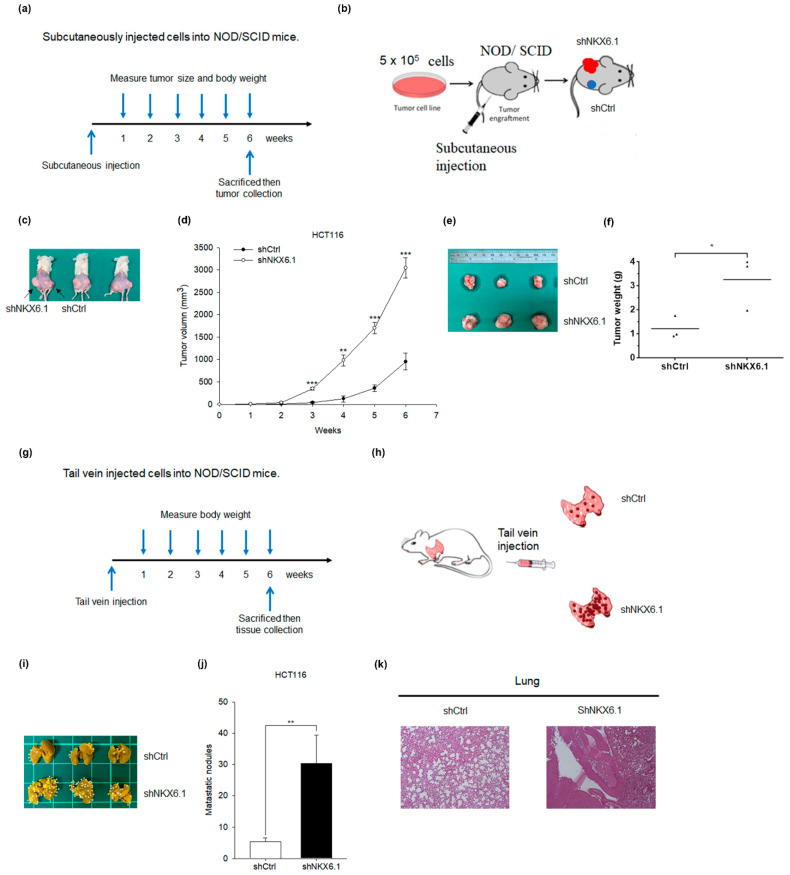
Knockdown of NKX6.1 promotes tumorigenesis and metastasis in xenograft mouse models. shNKX6.1-HCT116 or control shCtrl-HCT116 cells were subcutaneously injected into NOD/SCID mice. (**a**) The detailed manipulations of animal experiments are illustrated. (**b**) Schematic demonstrating the tumorigenesis assay model in vivo. The tumor growth and curve (**c**,**d**) and tumor weights and curve (**e**,**f**) of the shNKX6.1-HCT116 cells were compared with control shRNA (shCtrl). shNKX6.1-HCT116 or control shCtrl-HCT116 cells were injected into the tail vein of NOD/SCID mice. (**g**) The detailed manipulations of animal experiments are illustrated. (**h**) Schematic demonstrating the metastasis assay model in vivo. (**i**) The images are lungs excised from the mice; the arrows indicate lung nodules. (**j**) Lung nodule number. (**k**) The images are hematoxylin and eosin (H&E) staining of lungs (original magnification, ×200) from the mice. The values are expressed as the mean ± SD (analyzed by unpaired two-tailed *t*-test). * *p* < 0.05, ** *p* < 0.01, and *** *p* < 0.001.

To further confirm the effect of NKX6.1 on tumorigenicity in vivo, we subcutaneously injected shNKX6.1-HCT116 cells, in which NKX6.1 expression was knocked down by shRNAs targeting distinct coding sequences of NKX6.1 or control shCtrl-HCT116 cells into both flanks of NOD/SCID mice (Figure 5a,b). Six weeks after injection, the tumor mass was larger in the shNKX6.1 group than in the control group. The tumor growth rate was also increased in the shNKX6.1 group compared with the shCtrl control (Figure 5c–f). To further confirm the function of NKX6.1 in metastasis in vivo, we injected shNKX6.1-HCT116 cells or control shCtrl-HCT116 cells into mice via the tail vein (Figure 5g,h). Six weeks after injection, there were more metastatic lung nodules in the shNKX6.1 group than in the shCtrl control group (Figure 5i–k). Our data further confirmed that the knockdown of NKX6.1 promotes tumor growth and tumor metastasis in xenograft models.

### 2.5. NKX6.1 Represses Cancer Metastasis Partly through Inhibition of EMT

**Figure 6:**
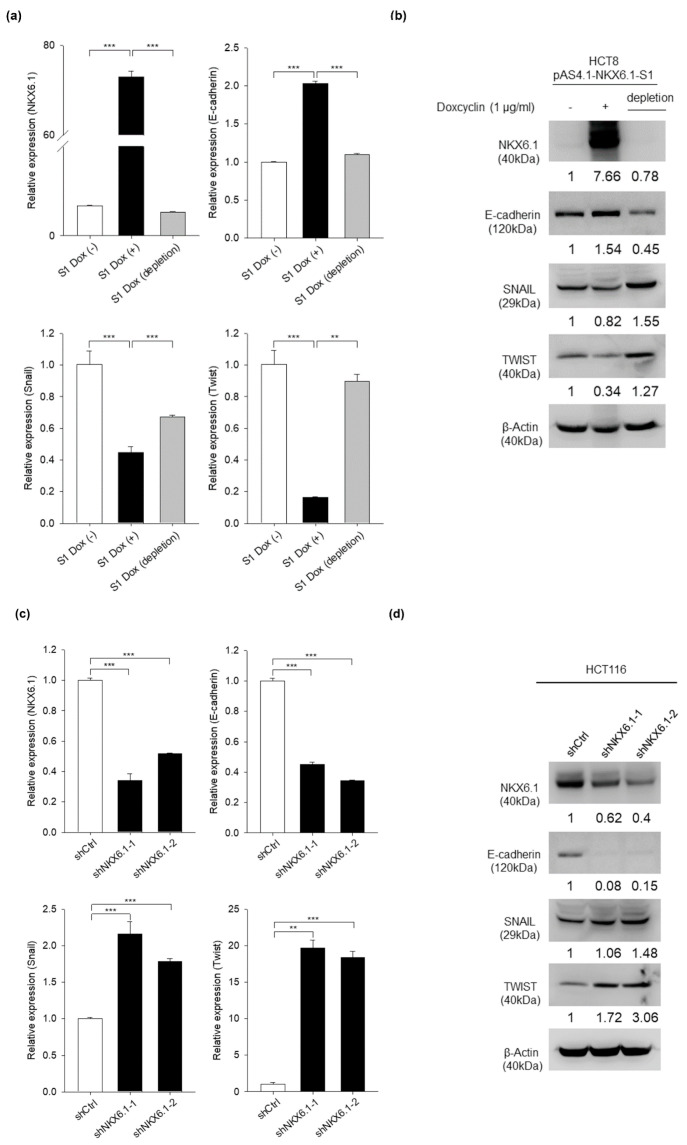
NKX6.1 represses cancer metastasis partly through inhibition of epithelial-to-mesenchymal transition (EMT). (**a**,**b**) The expression of E-cadherin and EMT regulators was detected by qRT-PCR and Western blotting in NKX6.1-expressing HCT8 cells. (**c**,**d**) The expression of E-cadherin and EMT regulators was detected by qRT-PCR and Western blotting in shNKX6.1-HCT116 cells. The values are expressed as the mean ± SD from three independent experiments (analyzed by unpaired two-tailed *t*-test). ** *p* < 0.01, and *** *p* < 0.001.

EMT plays important roles in the progression of primary tumors toward metastasis, which is likely caused by the activation of EMT-related transcription factors [42]. This critical process induces loss of cell-cell adhesion, as well as the loss of epithelial markers and the transition to mesenchymal characteristics [32,43,44,45]. In addition, our previous studies showed that NKX6.1 suppresses tumor metastasis ability through the epigenetic regulation of EMT in cervical cancer [20]. Therefore, we used inducible overexpression and knockdown strategies to explore whether NKX6.1 regulating EMT contributes to its metastasis suppressive function. We first analyzed the expression of SNAIL, TWIST, and E-cadherin to evaluate the effect of NKX6.1 on the EMT mechanism. In HCT8 cells, the ectopic expression of NKX6.1 repressed the mRNA and protein expression of SNAIL and TWIST, which are EMT-related transcription factors. In addition, NKX6.1 enhanced the expression of the epithelial marker E-cadherin (CDH1) at both the transcriptional and protein levels (Figure 6a,b) (Figures S5–S7). Moreover, the knockdown of NKX6.1 inhibited E-cadherin expression but enhanced SNAIL and TWIST at the mRNA and protein levels in HCT116 cells (Figure 6c,d) (Figures S8–10). Taken together, these results suggested that NKX6.1 could suppress tumor metastasis through the modulation of EMT-related transcription factors and EMT markers.

### 2.6. NKX6.1 Regulates Chemosensitivity to 5-Fluorouracil (5-FU) and Oxaliplatin in CRC Cell Lines

**Figure 7:**
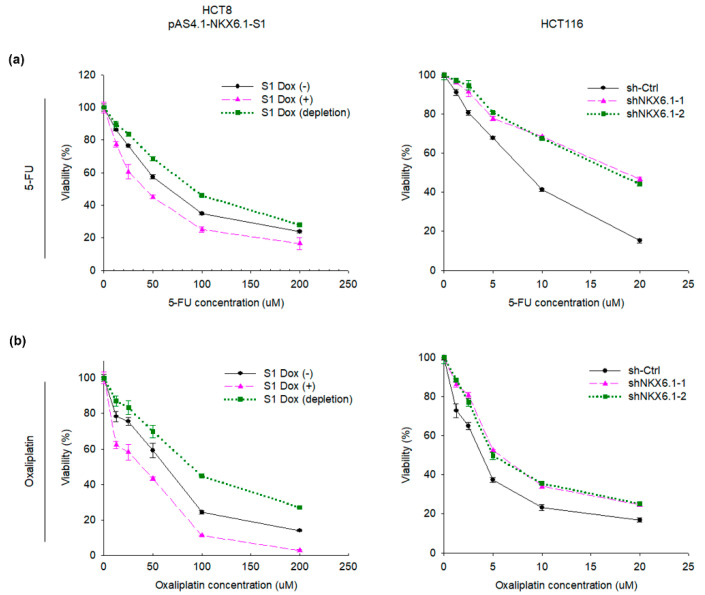
NKX6.1 regulates chemosensitivity to 5-fluorouracil (5-FU) and oxaliplatin in CRC cell lines. (**a**) NKX6.1-expressing HCT8 cells or NKX6.1-shRNA-knockdown HCT116 cells were cultured with 5-FU for 48 h and then assayed for cell viability; the line graphs are presented as the experiment/control cell ratio. (**b**) NKX6.1-expressing HCT8 cells or NKX6.1-shRNA-knockdown HCT116 cells were cultured with oxaliplatin for 48 h and then assayed for cell viability; the line graphs are presented as the experiment/control cell ratio. The data are shown as the mean ± SD from five independent experiments performed in triplicate.

More recently, we have demonstrated that the hypermethylation of *NKX6.1* may predict the outcome of stage II patients receiving chemotherapy, thus implying that NKX6.1 expression might be related to chemoresistance [29,30]. Considering the association of NKX6.1 with chemotherapy response, we then investigated whether NKX6.1 plays a role in the regulation of drug resistance in CRC cells. We determined the inhibitory effect of chemotherapy drugs (5-FU or oxaliplatin) in inducible NKX6.1-HCT8 cells and NKX6.1-knockdown-HCT116 cells using a cell viability assay. The cell viability of different cell groups was evaluated by a 3-(4,5-dimethylthiazol-2-yl)-5-(3-carboxymethoxyphenyl)-2-(4-sulfophenyl)-2H-tetrazolium (MTS) assay. The 50% inhibitory concentration (IC50) values of 5-FU or oxaliplatin in different groups of CRC cells are shown in Table 1. The results show that the overexpression of NKX6.1 significantly enhanced the sensitivity to chemotherapeutic drugs (5-FU or oxaliplatin) in HCT8 cells. Moreover, the knockdown of NKX6.1 notably attenuated sensitivity to chemotherapeutic drugs (5-FU or oxaliplatin) in HCT116 cells (Figure 7a,b). Collectively, these results suggested that NKX6.1 altered sensitivity to chemotherapeutic drugs (5-FU or oxaliplatin) in CRC cells.

### 2.7. Identification, Enriched Gene Ontology (GO) Functions and Kyuto Encyclopedia of Gene and Genomes (KEGG) Pathway Analysis of Differentially Expressed Genes (DEGs)

To further explore how NKX6.1 exerts its tumor-suppressive function, we used RNA-seq analysis to compare the differentially expressed genes in inducible NKX6.1-HCT8 cells after Dox treatment and Dox withdrawal. After normalization, EBSeq was used to screen the DEGs between control, NKX6.1-expressing, and NKX6.1-depleting HCT8 cells. In total, 1095 differentially expressed genes (posterior probability of being equally expressed (PPEE) < 0.05) were obtained; 172 genes were significantly upregulated (log_2_ FC > 1), and 79 genes were significantly downregulated (log_2_ FC < −1) (Table S2).

#### 2.7.1. Hierarchical Clustering of DEGs

**Figure 8:**
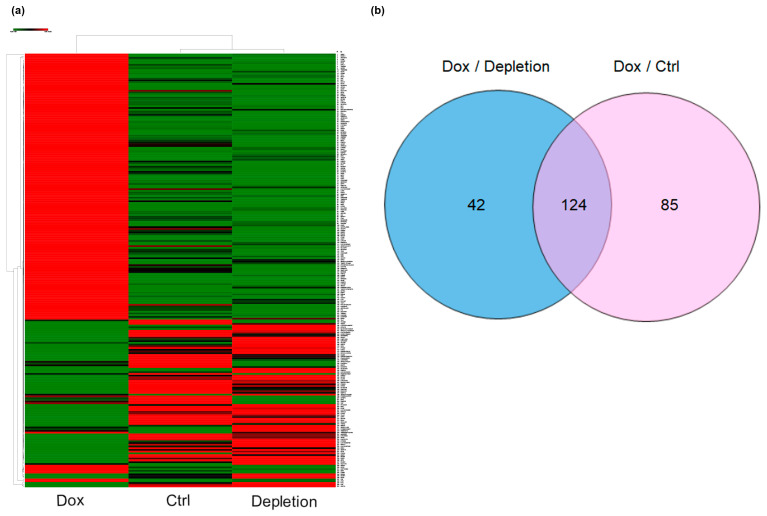

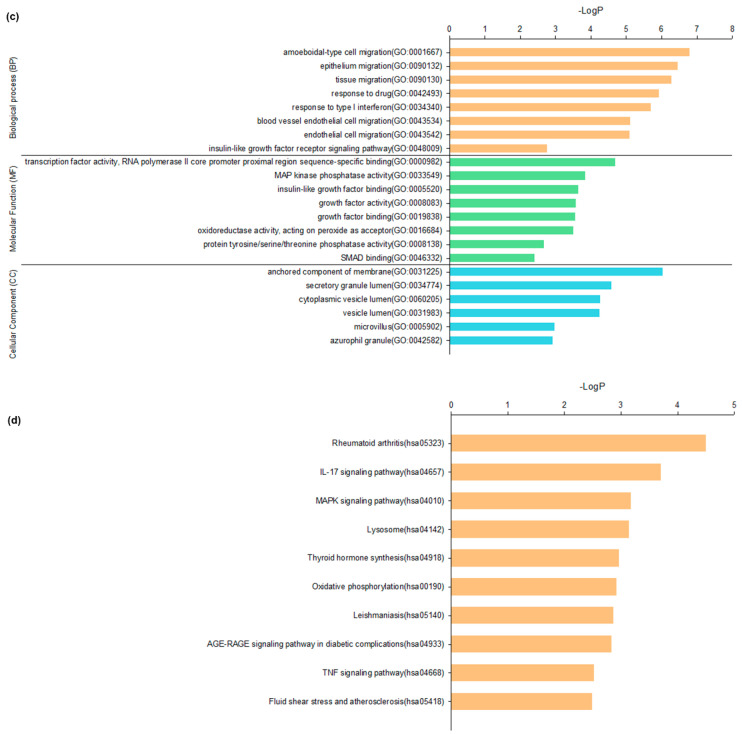
Identification, enriched Gene Ontology (GO) functions and Kyoto Encyclopedia of Genes and Genomes (KEGG) pathway analysis of differentially expressed genes (DEGs). (**a**) Cluster analysis of DEGs. The abscissa represents different samples; the vertical axis represents clusters of DEGs. Red represents upregulation; green represents downregulation. (**b**) Venn diagram analysis of DEGs. The blue circle represents the Dox induction V.S. depletion dataset, and the pink circle represents the Dox induction V.S. control dataset. The intersection of the two circles represents overlapping DEGs among the two datasets. DEGs, differentially expressed genes. (**c**) Enriched GO functions of DEGs. (**d**) KEGG pathway analysis of DEGs.

To assess whether the selected genes were distinguished well between control, NKX6.1-expressing, and NKX6.1-depleting HCT8 cells, we performed hierarchical clustering using Morpheus (https://software.broadinstitute.org/morpheus). In Figure 8a, rows correspond to genes, and columns correspond to samples. Based on the EBSeq differential gene expression (PPEE < 0.05), 251 differential genes were analyzed in the heatmap. The genes with similar expression patterns were clustered together. The upregulated genes are shown in red, and the downregulated genes are shown in green. Using Venn diagram analysis, 124 DEGs in the intersection of the above three groups were selected for further analysis (Figure 8b).

#### 2.7.2. GO Enrichment Analysis and KEGG Pathway Analysis of DEGs

The identified DEGs were uploaded to the online software Metascape for GO and KEGG pathway analyses. The results of the GO analysis revealed that 124 DEGs were significantly enriched in biological processes (BP), molecular function (MF), and cellular component (CC), including amoeboidal-type cell migration, epithelium migration, response to drug, blood vessel endothelial cell migration, transcription factor activity, MAP kinase phosphatase activity, growth factor activity, growth factor binding, protein tyrosine/serine/threonine phosphatase activity, SMAD binding, anchored component membrane and microvillus (Figure 8c).

KEGG pathway analysis revealed that the 124 DEGs were highly associated with pathways including the IL-17 signaling pathway, MAPK signaling pathway, lysosome, thyroid hormone synthesis, oxidative phosphorylation, AGE-RAGE signaling pathway, TNF signaling pathway, fluid shear stress, and atherosclerosis (Figure 8d). These results implied that these pathways might be related to the suppressive effect of NKX6.1 on CRC tumorigenesis, metastasis, and chemoresistance.

## 3. Discussion

NKX6.1 is an important transcription factor in the development of the pancreas and neurons [11,13,14,46]. In addition, our research team has reported that NKX6.1 inhibits metastasis through the epigenetic regulation of EMT in cervical cancer [20]. However, whether NKX6.1 plays important roles in CRC, EMT, and chemoresistance remains unclear. In this study, our data demonstrated that NKX6.1 has a critical role in suppressing tumorigenicity and metastatic behavior in vitro and in vivo models. Moreover, the overexpression of NKX6.1 alters chemosensitivity to 5-FU and oxaliplatin in CRC cells. RNA-seq data implied that NKX6.1 exerts its function by regulating many genes related to cell migration, response to drug, and growth factor activity. Taken together, our results demonstrated that NKX6.1 could suppress tumor metastasis by repressing EMT-related transcription factors and EMT markers and enhance chemosensitivity in CRC cells.

In our previous studies, we demonstrated that NKX6.1 is a bifunctional transcription factor that suppresses EMT in cervical cancer cells by increasing the expression of the epithelial marker E-cadherin and decreasing the expression of the mesenchymal marker vimentin [20]. NKX6.1 did not regulate EMT-related transcription factors but directly regulated epithelial markers and mesenchymal markers in cervical cancer. Therefore, we hypothesized that NKX6.1 functions as a suppressor in the tumor development and metastasis of CRC. Here, we confirmed that NKX6.1 repressed tumorigenesis and metastasis in CRC cell lines and xenograft models. However, our data demonstrated that NKX6.1 could repress the expression of SNAIL and TWIST at the transcriptional and protein levels in CRC cells. Moreover, using the Eukaryotic Promoter Database (EPD) (http://epd.vital-it.ch), we discovered five putative NKX6.1 binding sites in the promoter of 2.5 kb of *SNAIL* as well as one putative NKX6.1 binding site in the promoter of 2.5 kb of *TWIST.* The suppressive effect on EMT might be mediated through the EMT-related transcription factors SNAIL and TWIST. Nevertheless, whether NKX6.1 directly regulates EMT-related markers needs further investigation.

In addition to our reports, the abnormal methylation of *NKX6.1* was frequently observed in many cancers, including B-cell lymphoma [24], acute lymphoblastic leukemia [25], astrocytoma [26], and gastric cancer [27,28]. Based on these papers, *NKX6.1* could be a potential biomarker and tumor suppressor gene in cancers. However, Huang et al. reported that the upregulation of NKX6.1 is notably correlated with cancer progression and predicts unfavorable prognosis in hepatocellular carcinoma (HCC) [47]. More recently, Li and his colleagues reported that NKX6.1 is a factor for IL- 6-regulated growth and tumor formation in basal-like breast cancer [48] (Table S3). *NKX6.1* might function as an oncogene in these cancers. This evidence suggests that NKX6.1 likely has a different functional role in different types of cancer.

Moreover, we recently demonstrated that *NKX6.1* methylation was an independent indicator of 5-year disease-free survival in stage II CRC patients receiving adjuvant chemotherapy [29,30]. These data suggested that NKX6.1 may be a possible target of resistance to chemotherapy in aggressive tumors. Identification of the potential therapeutic targets for chemoresistance is important to improve the outcome of CRC patients. In the present study, we demonstrated that NKX6.1 may increase the drug sensitivity of 5-FU and oxaliplatin in cell models. Furthermore, the EMT mechanism plays important roles in organ fibrosis, therapeutic resistance, and metastatic dissemination [32,33,49,50]. These data implied that EMT might mediate the function of NKX6.1 in increasing chemosensitivity. However, the underlying regulatory mechanisms are complex due to the tumor microenvironment and require further investigation.

To further investigate the downstream signaling pathways associated with the tumor suppressor function of NKX6.1, we used RNA sequencing technology for comprehensive analysis. Many pivotal genes and pathways associated with cancer were identified in the present study. The results of GO function annotation showed that DEGs were mainly related to cell migration, response to drug, transcription factor activity, and growth factor activity, and suggested that these DEGs are involved in the function of NKX6.1 suppressing cancer invasion and metastasis. Accumulating evidence suggests that CRC cells interact with stoma cells by producing extracellular matrix (ECM) components, mediating direct cell-cell contact and secreting growth factors [51]. However, many genes and signal pathways are predicted to be associated with the function of NKX6.1. To further clarify the downstream signals regulated by NKX6.1, RNA sequencing data of different CRC cells should be simultaneously analyzed to identify significant pathways.

In summary, we found that NKX6.1 suppresses CRC cell invasion by inhibiting EMT. Furthermore, our data demonstrated that chemosensitivity is enhanced in CRC cell lines through NKX6.1 overexpression. Moreover, the RNA-seq data revealed that the function of NKX6.1 is associated with DEGs including cell migration, angiogenesis, response to drug, anchored component of membrane, transcription factor activity, MAP kinase phosphatase activity, growth factor activity, and growth factor binding.

## 4. Materials and Methods

### 4.1. Cell Culture

HT29 cells were purchased from the American Type Culture Collection. HCT8, HCT116, SW480, and SW620 cells were purchased from the Food Industry Research and Development Institute (Taiwan). Cells were cultured in Roswell Park Memorial Institute (RPMI) 1640 medium (GIBCO, Gaithersburg, MD, USA) supplemented with 2 mM L-glutamine, 1% nonessential amino acids (NEAA), 10% heat-inactivated fetal bovine serum (FBS), 100 U/mL penicillin and 100 μg/mL streptomycin (GE Healthcare Life Sciences, Chicago, IL, USA). All cell cultures were grown as monolayer cultures and maintained in a humidified atmosphere containing 5% CO_2_ in air at 37 °C.

### 4.2. Plasmids, shRNA Clones and Constructs

The NKX6.1 full-length open-reading frame (ORF) cDNA product was constructed into the pLAS2.1w.PeGFP-I2-Bsd constitutive expression vector (National RNAi Core Facility, Taipei, Taiwan) (termed pLAS2.1-NKX6.1) or the inducible expression vector pAS4.1w. Pbsd-aOn (National RNAi Core Facility, Taipei, Taiwan) (termed pAS4.1-NKX6.1). pLKO.1-shLacZ and shNKX6.1 (National RNAi Core Facility, Taipei, Taiwan.) are described in Table S1.

### 4.3. Generation of Cells Overexpressing Stable or Inducible Transfects or Stable Knockdown Expression

Assays for the generation of cells overexpressing stable or inducible transfects or with stable knockdown expression were conducted as described previously [20,52].

### 4.4. Lentivirus Production and Infection

Assays for lentivirus production and infection were conducted as described previously [53].

### 4.5. Real-Time PCR and Immunoblotting

Assays for real-time PCR and immunoblotting were conducted as described previously [53,54]. The primers used in real-time PCR are shown in Supplementary Table S2. The following primary antibodies were used in the immunoblotting assay: anti-NKX6.1 (AF5857; R&D systems, Minneapolis, MN, USA), anti-E-cadherin (610405; BD Biosciences, San Jose, CA, USA), anti-vimentin (sc-6260; Santa Cruz Biotechnology, Santa Cruz, CA, USA), anti-SNAIL (GTX100754; GeneTex, Irvine, CA, USA), anti-TWIST (ab49254; Abcam, Cambridge, MA, USA), and anti-SLUG (#9585; Cell Signaling, Danvers, MA, USA). Horseradish peroxidase-conjugated rabbit anti-mouse or goat anti-rabbit secondary antibodies (Santa Cruz Biotechnology, Santa Cruz, CA, USA) were used as appropriate.

### 4.6. Assays for Cell Viability, Anchorage-Independent Growth (AIG) and Invasion Assay

Assays for cell viability by MTS, anchorage-independent growth (AIG), and invasion were conducted as described previously [55].

### 4.7. In Vivo Tumor Xenograft and Metastasis Model

Six-week-old NOD/SCID female mice were used in the tumorigenicity and metastasis analysis. All animal studies were approved by the Institutional Animal Care and Use Committee of the National Defense Medical Center (Taipei, Taiwan). The use of these animal were approved by our Institutional Review Board (IACUC No: 19-096). Detailed tumor xenograft and metastasis analyses were conducted as described previously [55,56].

### 4.8. Drugs

Doxycycline was obtained from MilliporeSigma (MilliporeSigma, Burlington, MA, USA) oxaliplatin was obtained from Zydus Hospira Oncology Private Limited (Matoda, India) and 5-FU was obtained from Nang Kuang Pharmaceutical Co. Ltd. (Taipei, Taiwan). Doxycycline, oxaliplatin, and 5-FU were dissolved in distilled water. Aliquots were stored at −20 °C for up to a maximum of 3 months and thawed immediately before use.

### 4.9. RNA Sequencing Data Analysis

#### 4.9.1. Quality Control, Read Mapping and Counting Reads in Features

Quality control and preprocessing of FASTQ files are essential in providing clean data for downstream analysis. By filtering raw reads using Trimmomatic (http://www.usadellab.org/cms/?page=trimmomatic), high-quality data were obtained. To filter out low-quality sequences, sequences were trimmed to more than 30% of reads with quality less than Q20 and adaptors (first 12 bp of reads). Then, the clean reads were acquired and then aligned against the reference genome with reference annotation (Aligner: bowtie2, Reference: transcript sequences). The Homo sapiens hg19 genome reference and annotation GTF file was downloaded from the UCSC Genome Browser (https://genome.ucsc.edu/cgi-bin/hgGateway). Using bowtie2 (http://bowtie-bio.sourceforge.net/bowtie2/index.shtml), all clean reads were mapped to the Homo sapiens genome (mapping efficiencies (95%) for each paired end read). Then, for the reading of transcripts, counting was performed with RSEM (http://deweylab.github.io/RSEM/EBSeq). Furthermore, using EBSeq (https://www.biostat.wisc.edu/~kendzior/EBSEQ/), differential expression analysis was performed on three replicates of each sample. Student’s t-test was used to identify DEGs with an alteration of ≥2-fold. *p* < 0.05 was considered a statistically significant difference.

#### 4.9.2. GO and Pathway Enrichment Analysis of DEGs

GO analysis and KEGG pathway analysis were conducted to identify DEGs at the biologically functional level. The Metascape database (https://metascape.org/gp/index.html#/main/step1) for annotation, visualization, and integrated discovery was used to integrate functional genomic annotations. *p* < 0.05 was considered to indicate a statistically significant difference.

### 4.10. Statistical Analysis

The statistical analyses were performed using GraphPad Prism software (version 4.03; GraphPad Software, La Jolla, CA, USA) and SPSS software (IBM SPSS Statistics 21; Asia Analytics Taiwan, Taipei, Taiwan). The quantitative data are presented as the mean ± standard deviation (SD). Statistical comparisons between two groups were performed using the unpaired two-tailed t-test. In all cases, *p* < 0.05 was considered statistically significant.

## 5. Conclusions

Taken together, our results demonstrated that NKX6.1 functions as a tumor suppressor partly by repressing EMT and enhancing chemosensitivity in CRC, making it a potential therapeutic target.

## Figures and Tables

**Table 1:** Estimated 50% inhibitory concentration (IC50) in cultured CRC cell lines.

		Estimated IC_50_ Value	
Cultured Cell Line	Treatment	5-Fluorouracil (μM)	Oxaliplatin (μM)
HCT8 (pAS4.1-NKX6.1-S1)	Dox (–)	61.95	49.84
	Dox (+)	41.41	28.55
	Dox (depletion)	81.17	78.74
HCT116 (shCtrl)		6.93	3.86
HCT116 (shNKX6.1-1)		13.24	6.19
HCT116 (shNKX6.1-2)		13.58	6.12

The 50% inhibitory concentration (IC50) values were estimated from the log concentration effect curves and nonlinear regression analysis.

## Supplementary Materials

Supplementary materials can be found at https://www.mdpi.com/1422-0067/21/14/5106/s1. Figure S1. Protein expression of NKX6.1 in HCT8, HT29, SW480 and SW620 cell lines. Figure S2. Protein expression of NKX6.1 in constitutive NKX6.1-HCT8. Figure S3. Protein expression of NKX6.1 in inducible NKX6.1-HCT8. Figure S4. Protein expression of NKX6.1 in NKX6.1-knockdown-HCT116. Figure S5. Protein expression of E-cadherin (CDH1) in inducible NKX6.1-HCT8. Figure S6. Protein expression of SNAIL in inducible NKX6.1-HCT8. Figure S7. Protein expression of TWIST in inducible NKX6.1-HCT8. Figure S8. Protein expression of E-cadherin in NKX6.1-knockdown-HCT116. Figure S9. Protein expression of SNAIL in NKX6.1-knockdown-HCT116. Figure S10. Protein expression of TWIST in NKX6.1-knockdown-HCT116. Table S1. Sequence of the oligonucleotides for making the shRNA constructs, real-time PCR. Table S2. 251 Differentially expressed genes (DEGs). Table S3. NKX6.1 has a different functional role in different types of cancer.
