# Supplementary material for: NKX6.1 Represses Tumorigenesis, Metastasis, and Chemoresistance in Colorectal Cancer
Source: Int J Mol Sci. 2020 Jul 19;21(14):5106. doi: 10.3390/ijms21145106 (PMC7404324; doi:10.3390/ijms21145106)
Supplement: Supplementary file 1 [file ijms-21-05106-s001.zip › Table S3.docx]

Table S3. NKX6.1 has a different functional role in different types of cancer

| Cancer type | Technique used | Notable finding | Reference |
| --- | --- | --- | --- |
| B-cell lymphoma | Microarray, Methylation-specific PCR (MSP), Quantitative real-time MSP (QMSP) | NKX6.1 is hypermethylation. | Leukemia 2006, 20, 1855-1862 |
| Acute lymphoblastic leukemia | Microarray, MSP, QMSP | NKX6.1 is hypermethylation. | Cancer Res 2007, 67, 2617-2625 |
| Astrocytoma | Microarray, Bisulfite sequencing | NKX6.1 is hypermethylation. | Cancer Res 2010, 70, 2718-2727 |
| Gastric cancer | Pyrosequencing | NKX6.1 is hypermethylation. | Cancer Epidemiol Biomarkers Prev 2015, 24, 1607-1613 |
| Gastric cancer | QMSP | NKX6.1 is hypermethylation. | Gut 2015, 64, 388-396 |
| Hepatocellular carcinoma | QPCR, Western blot, Cell invasion assay, MTT, Immunocytochemistry (IHC) | NKX6.1 is up-regulated. | Tumour biol 2015, 36, 4405-4415 |
| Basal-like breast cancer | QPCR, Western blot, Growth assay, IHC, TUNEL assay, Luciferase assay, Pull-down assay, Chromatin immunoprecipitation (ChIP) assay, Electrophoretic mobility shift assay (EMSA), animal experiment | NKX6.1 is up-regulated. | Experimental cell research 2016, 343, 177-189 |
