# Supplementary figures and images for: NKX6.1 Represses Tumorigenesis, Metastasis, and Chemoresistance in Colorectal Cancer
Source: Int J Mol Sci. 2020 Jul 19;21(14):5106. doi: 10.3390/ijms21145106 (PMC7404324; doi:10.3390/ijms21145106)

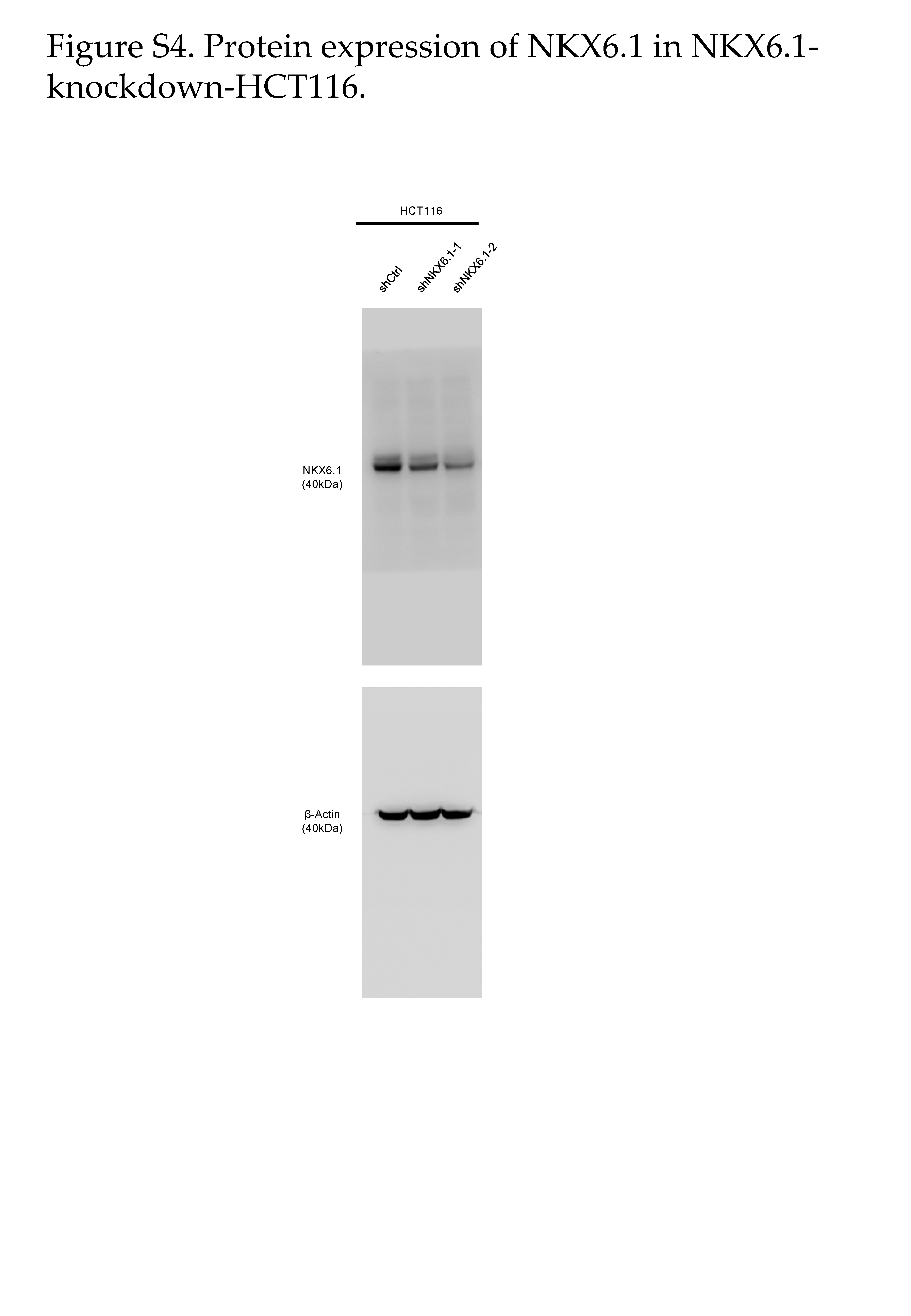

Supplement: Supplementary file 1 [file ijms-21-05106-s001.zip › Figure S4.tiff]

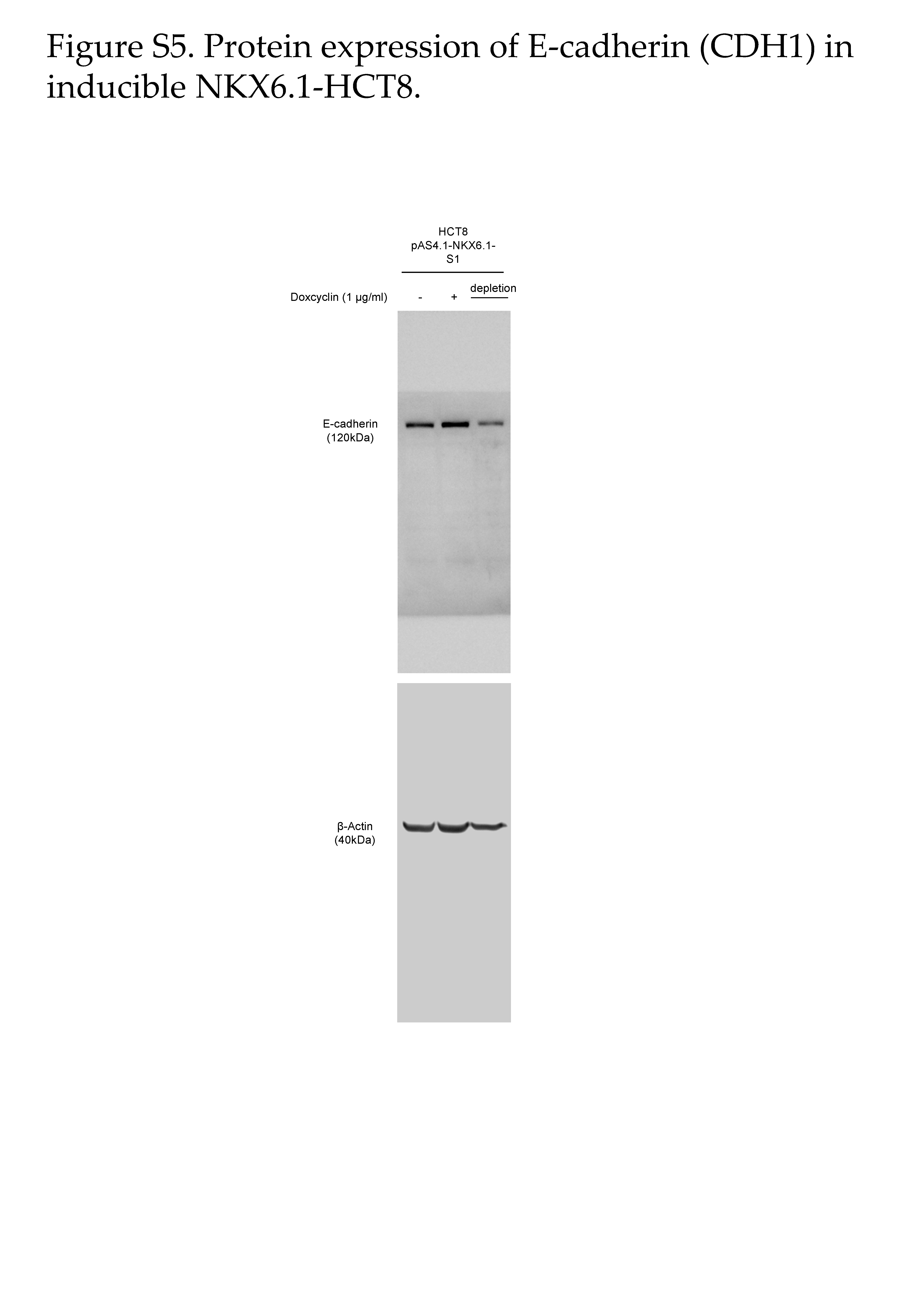

Supplement: Supplementary file 1 [file ijms-21-05106-s001.zip › Figure S5.tiff]

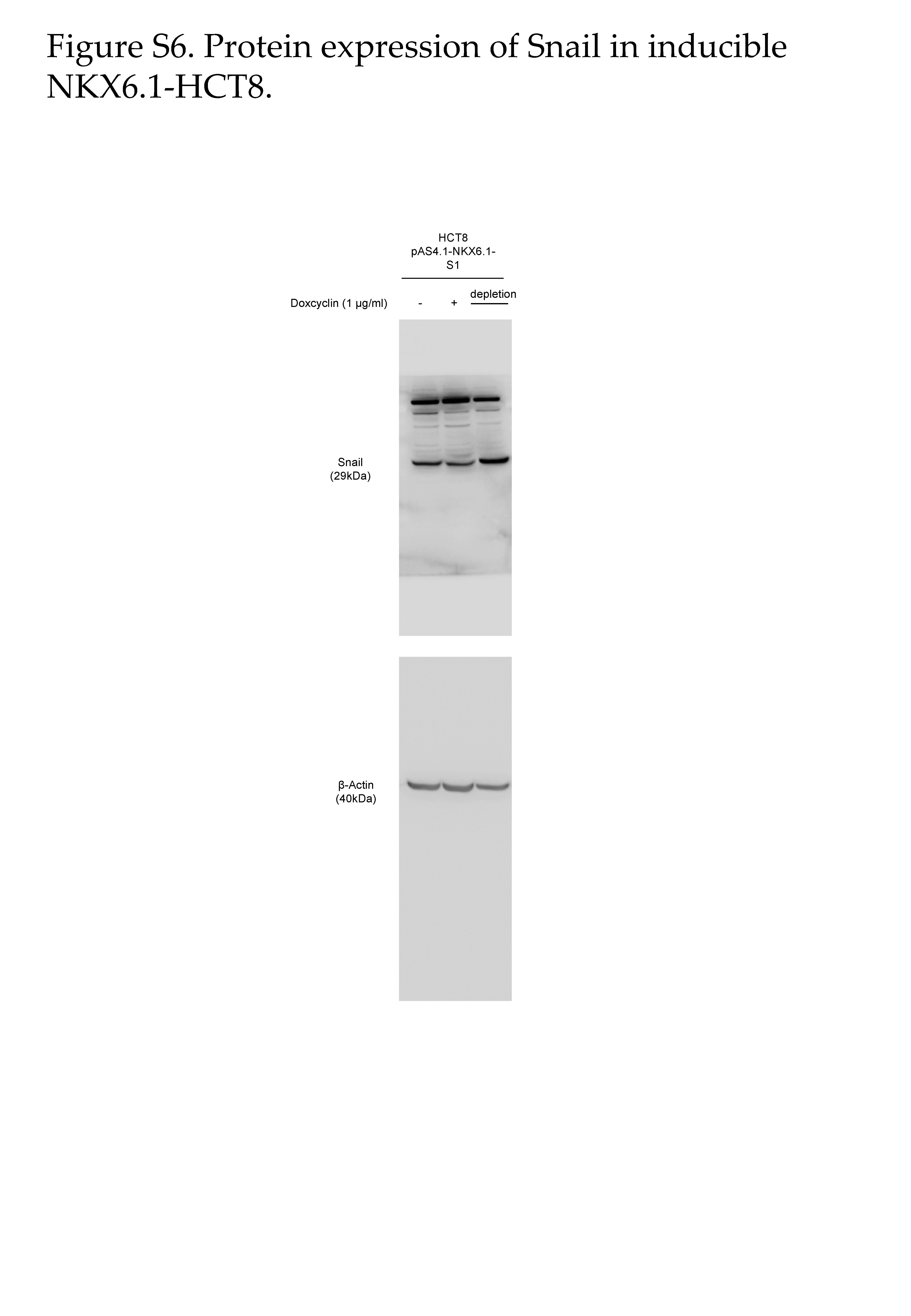

Supplement: Supplementary file 1 [file ijms-21-05106-s001.zip › Figure S6.tiff]

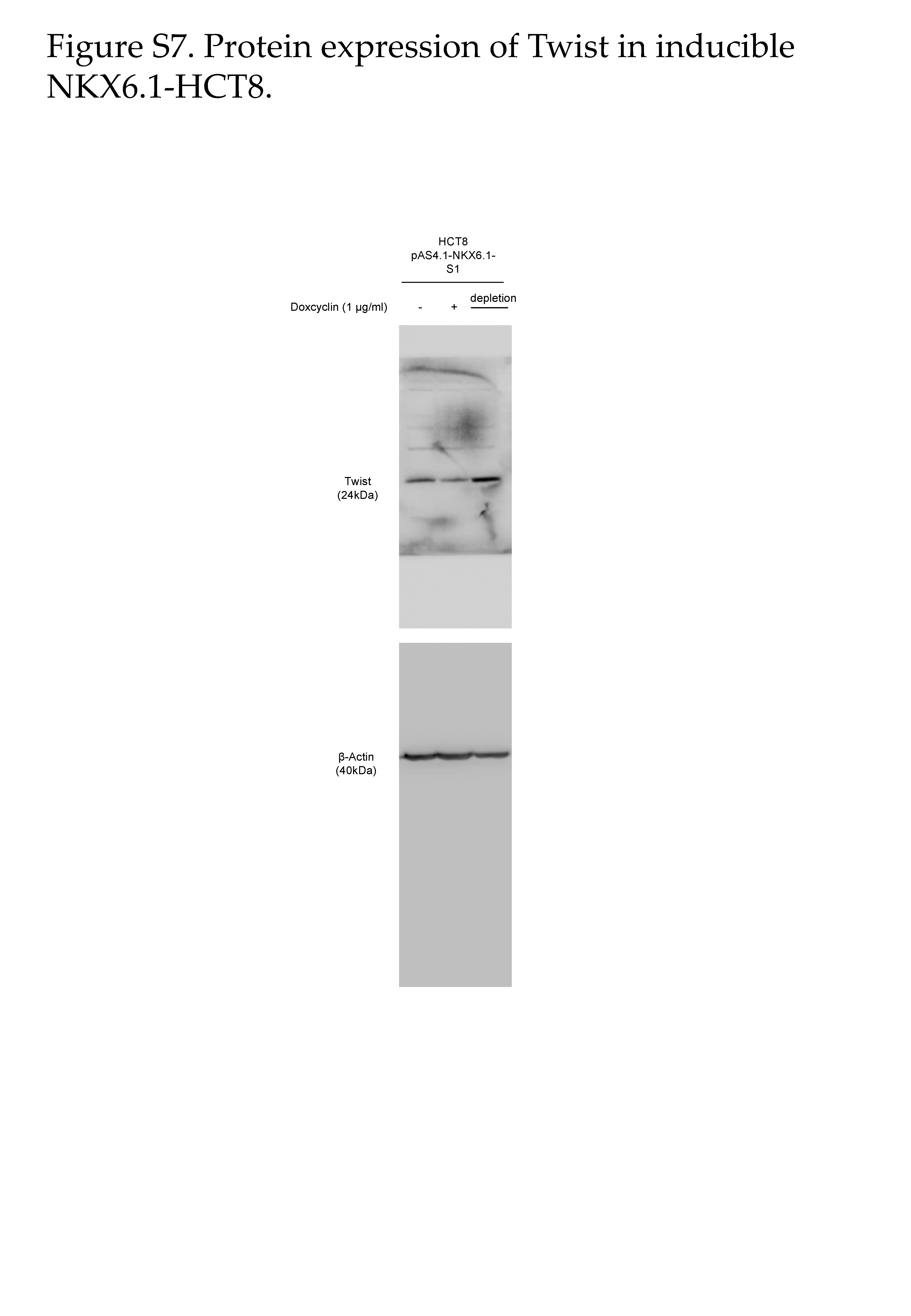

Supplement: Supplementary file 1 [file ijms-21-05106-s001.zip › Figure S7.tiff]

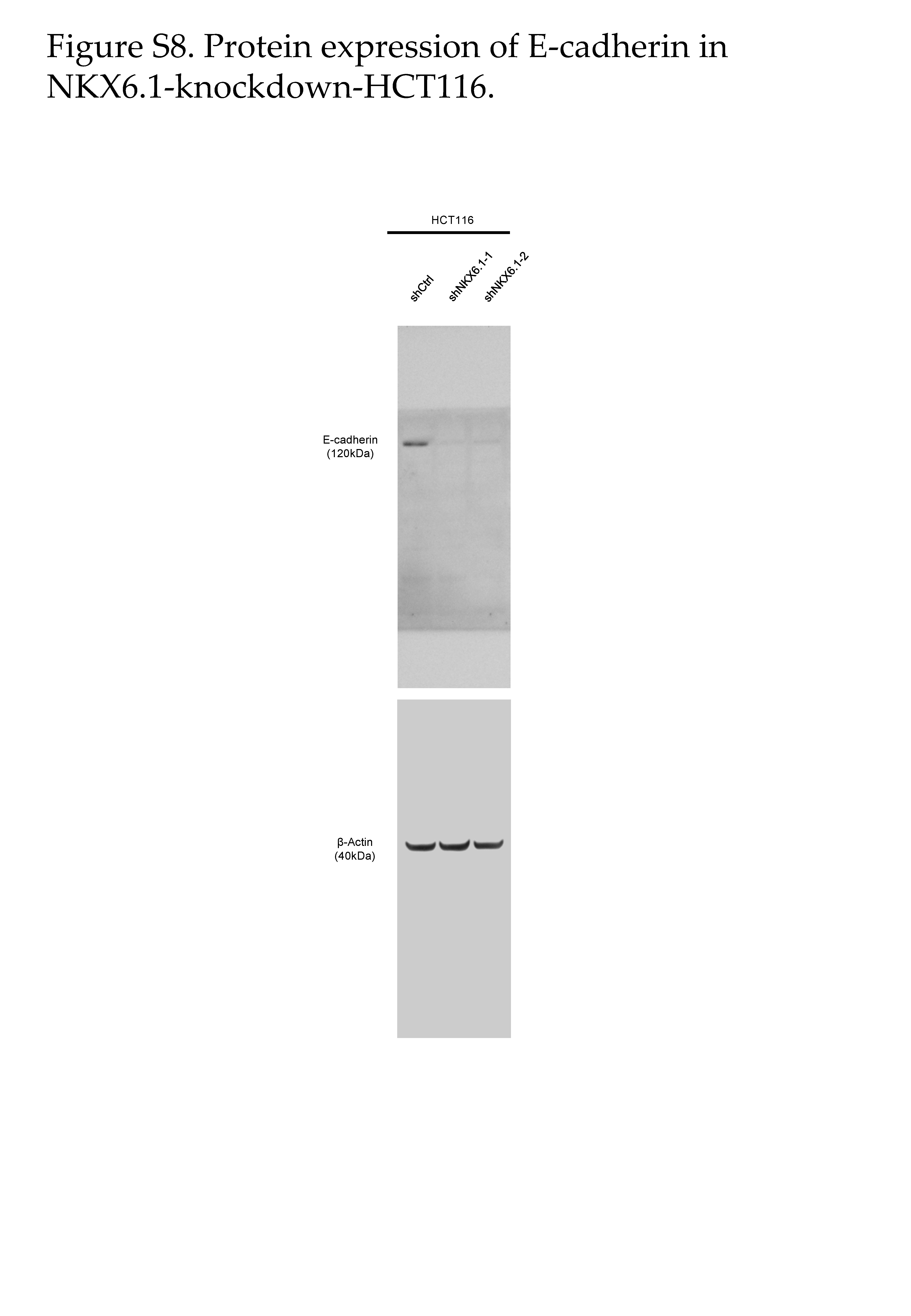

Supplement: Supplementary file 1 [file ijms-21-05106-s001.zip › Figure S8.tiff]

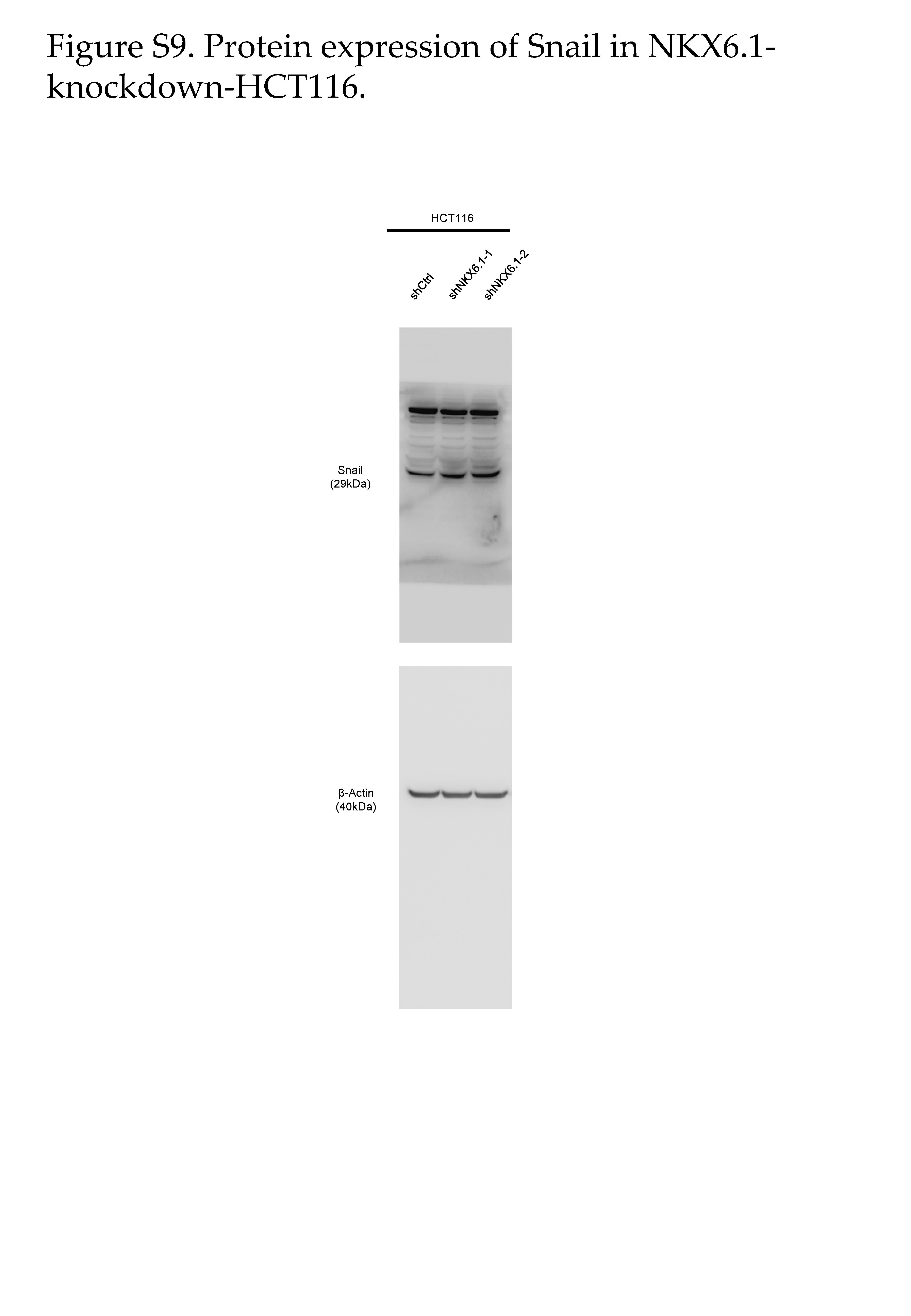

Supplement: Supplementary file 1 [file ijms-21-05106-s001.zip › Figure S9.tiff]

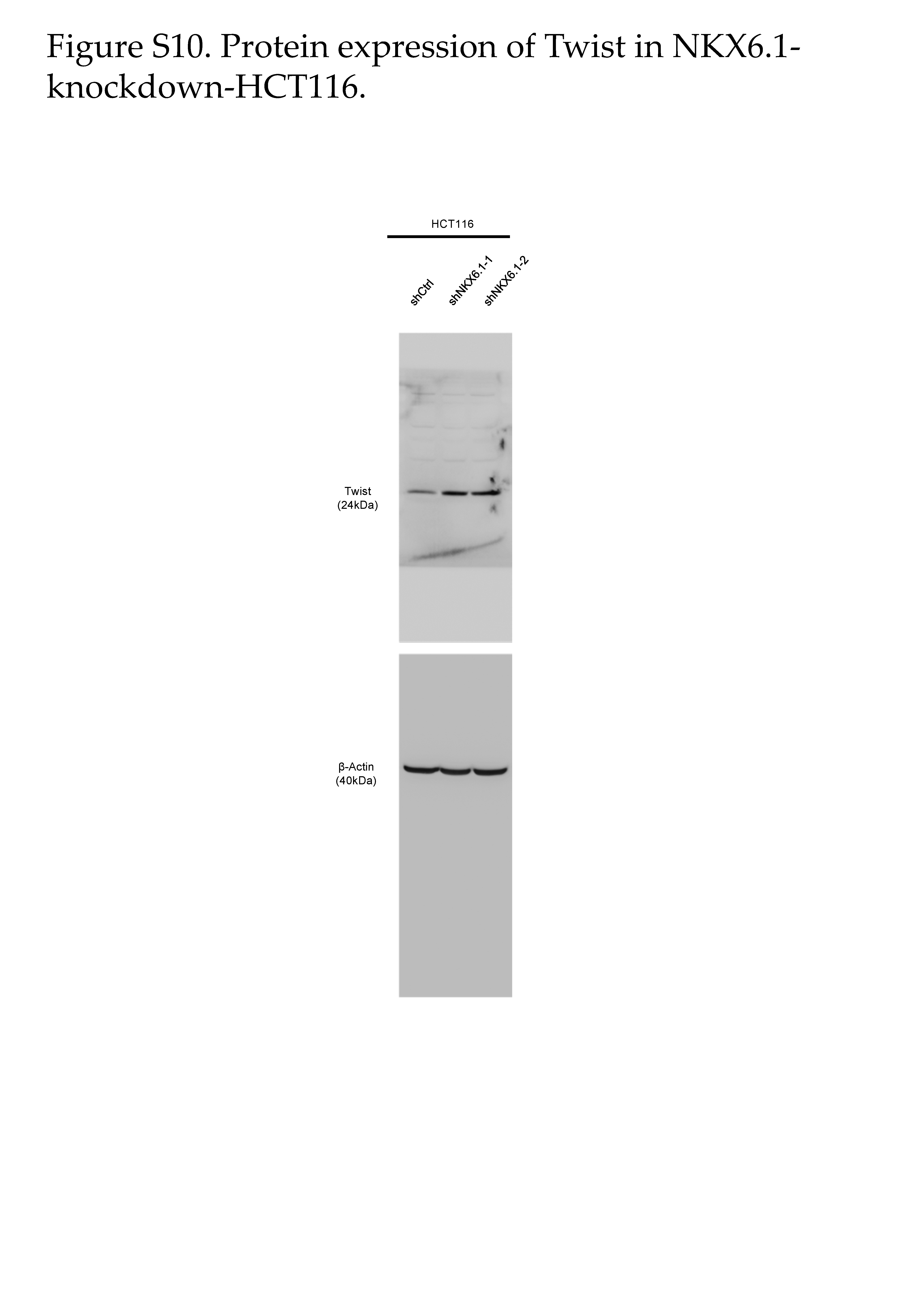

Supplement: Supplementary file 1 [file ijms-21-05106-s001.zip › Figure S10.tiff]

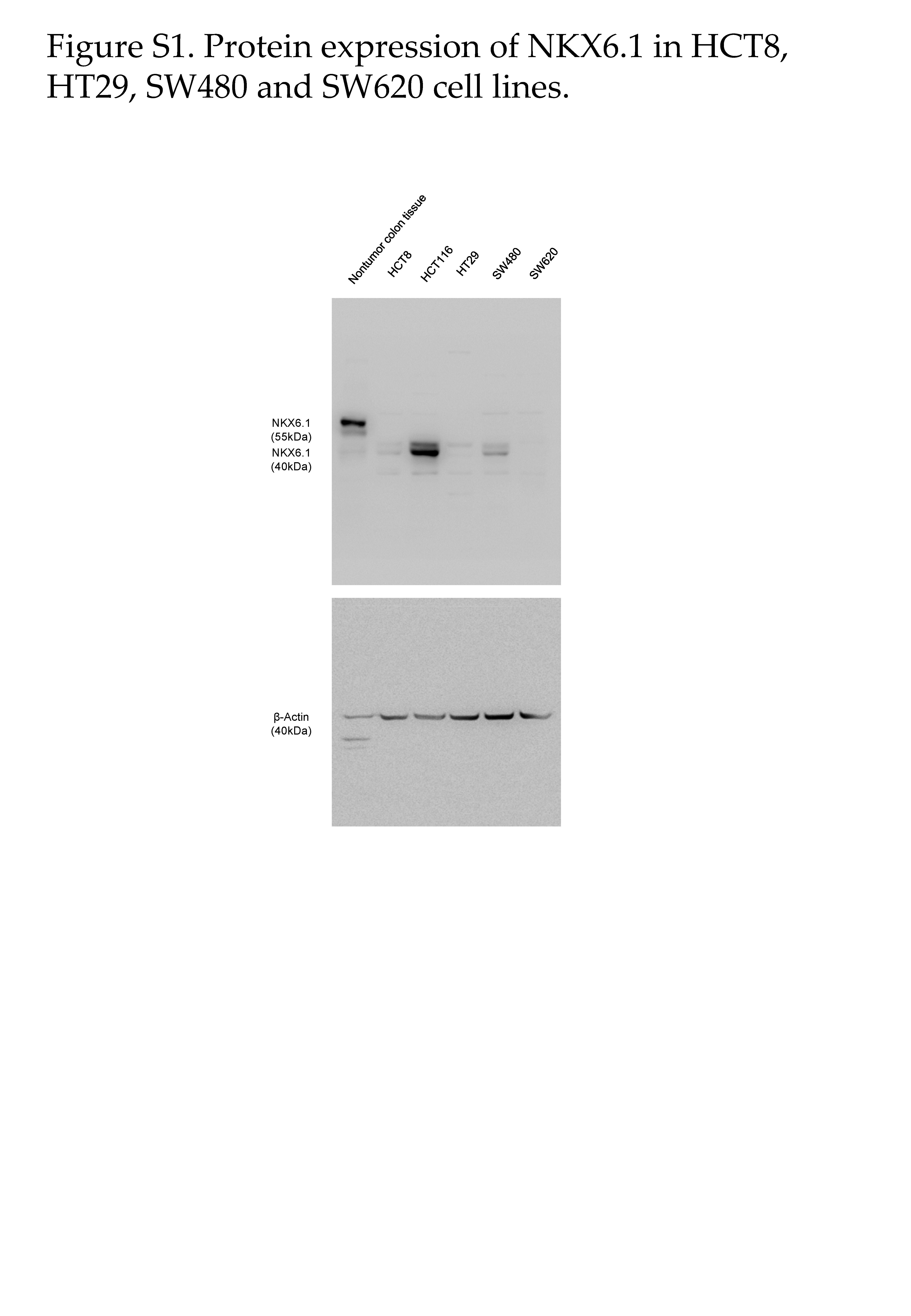

Supplement: Supplementary file 1 [file ijms-21-05106-s001.zip › Figure S1.tiff]

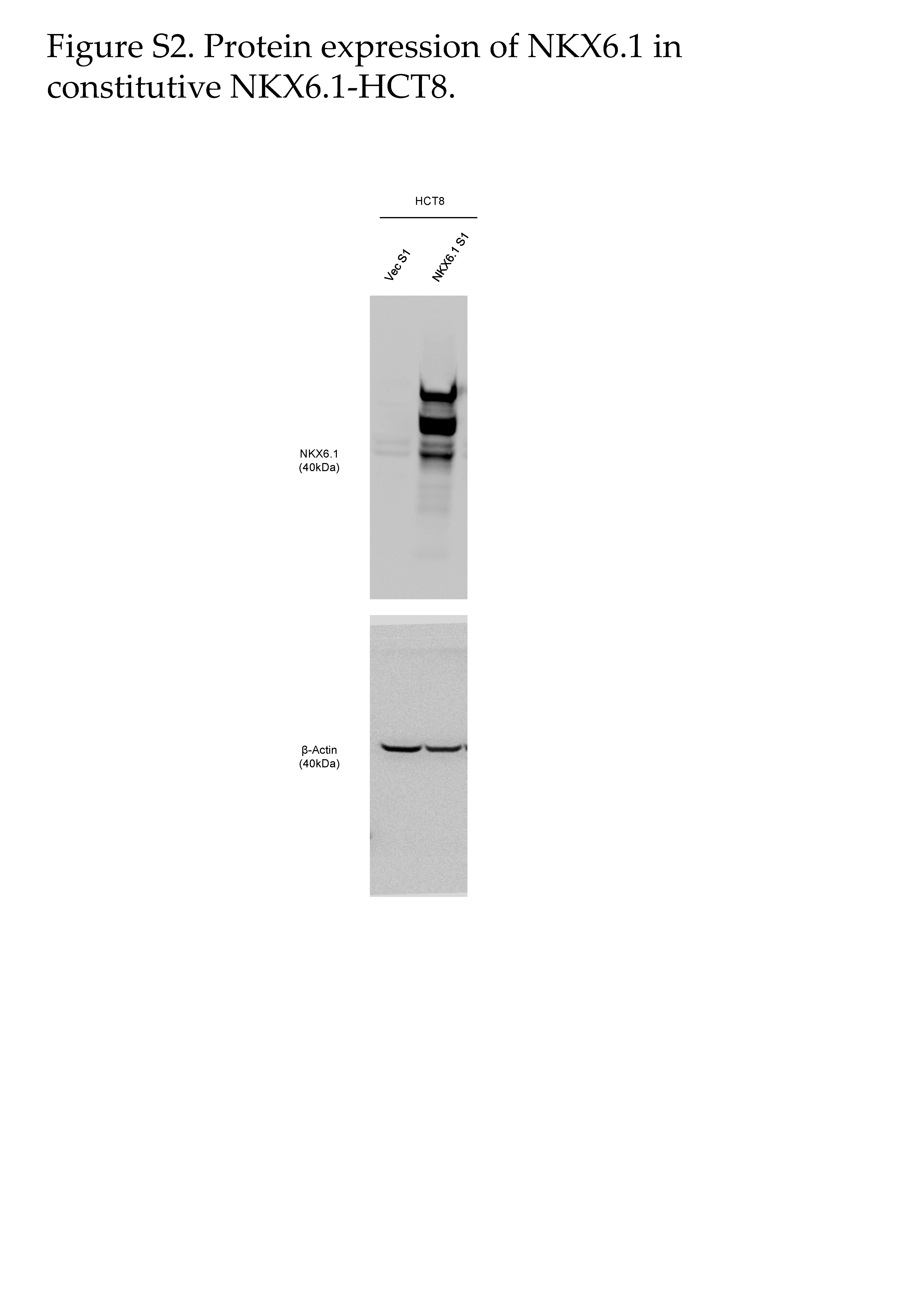

Supplement: Supplementary file 1 [file ijms-21-05106-s001.zip › Figure S2.tiff]

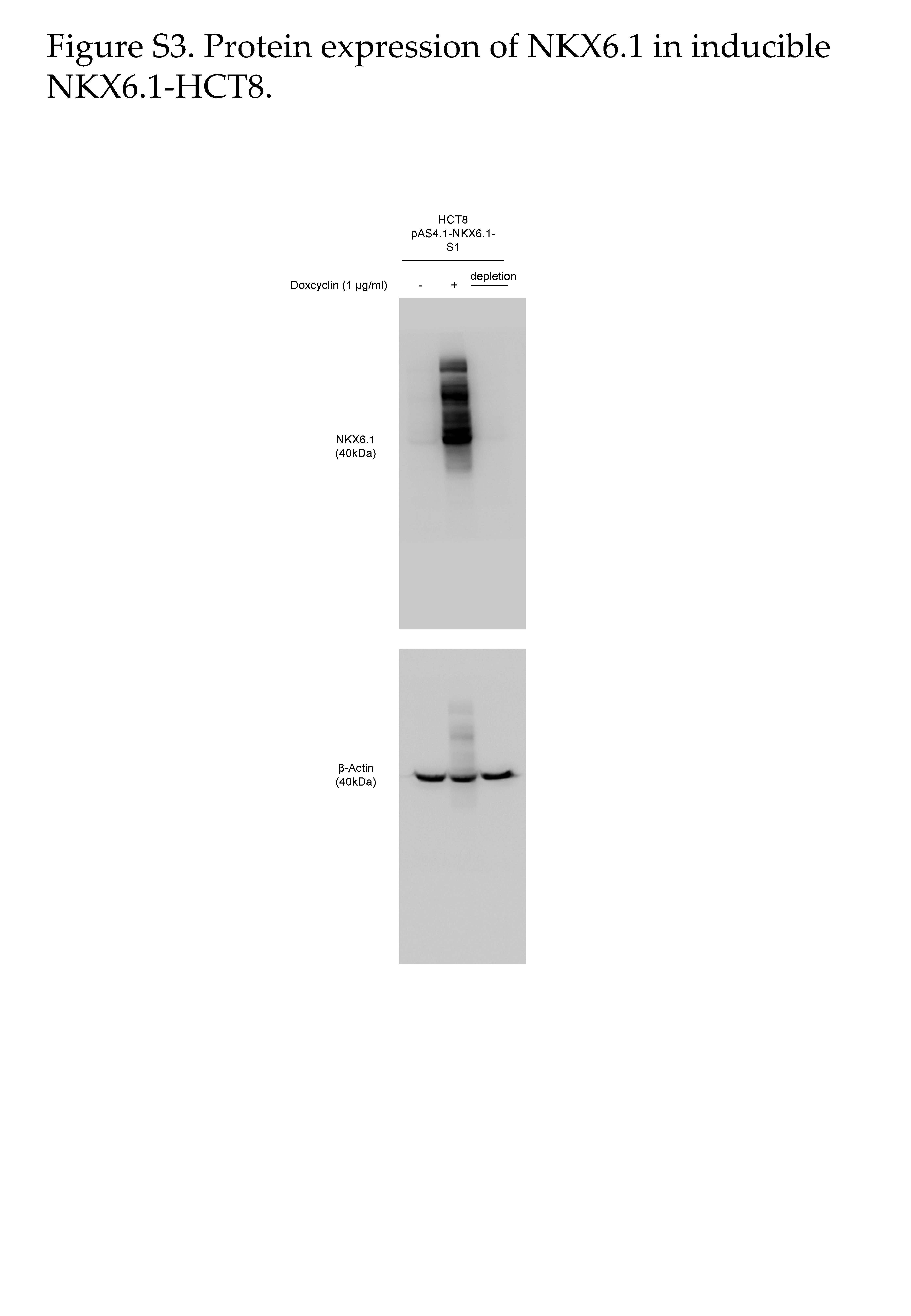

Supplement: Supplementary file 1 [file ijms-21-05106-s001.zip › Figure S3.tiff]
